# Impact of Endothelial Diversity and Dysfunction on Cardiovascular Disease

**DOI:** 10.1002/cph4.70064

**Published:** 2025-11-02

**Authors:** Anna E. Williams, Luke S. Dunaway, Zuzanna J. Juśkiewicz, Wyatt J. Schug, Miriam M. Cortese‐Krott, Michael Koval, Brant E. Isakson

**Affiliations:** ^1^ Division of Pulmonary, Allergy, Critical Care and Sleep Medicine, Department of Medicine Emory University School of Medicine Atlanta Georgia USA; ^2^ Department of Cell Biology Emory University School of Medicine Atlanta Georgia USA; ^3^ Robert M. Berne Cardiovascular Research Center University of Virginia School of Medicine Charlottesville Virginia USA; ^4^ Department of Molecular Physiology and Biophysics University of Virginia School of Medicine Charlottesville Virginia USA; ^5^ Myocardial Infarction Research Laboratory, Department of Cardiology, Pulmonology, and Angiology, Medical Faculty Heinrich‐Heine‐University Düsseldorf Germany; ^6^ Department of Physiology and Pharmacology Karolinska Institute Stockholm Sweden

## Abstract

Endothelial cells (ECs) are functionally heterogeneous, even in vascular beds within the same organ. As the key cell type lining the vascular lumen, ECs regulate vascular tone (control of blood vessel diameter), permeability of molecules, water, and ions across the vascular wall, vessel composition through cell–cell contacts, regulation of tissue redox status, and cytokine signaling. ECs are also influenced by mechanical stimuli such as blood flow. Many of these features can be analyzed in multi‐cellular in vitro models that provide a controlled setting to investigate EC biology. Endothelial dysfunction (ED) has emerged as a central pathophysiological mechanism connecting different underlying cardiovascular diseases. Given that ECs are functionally heterogeneous, developing novel therapeutical approaches that target EC subtypes in specific tissues is anticipated to provide new diagnostic markers and therapeutic approaches for organ‐specific treatment of cardiovascular disease.

## Graphical abstract

The vascular endothelium is highly diverse, regulating permeability, vascular tone, repair, and inter‐organ communication. Endothelial heterogeneity supports tissue‐specific needs, but dysfunction drives cardiovascular disease. Transcriptomics, in vitro models, and redox signaling studies reveal therapeutic targets for precision treatment of endothelial dysfunction.

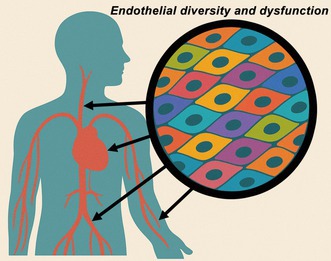

## 1 Functional and Phenotypic Diversity of the Vascular Endothelium

The cardiovascular system forms a network that serves the metabolic needs of tissues by delivery of oxygen and removal of carbon dioxide via circulating erythrocytes (Poole et al. 2021). Tissue homeostasis is also served by nutrient delivery and removal of waste products by blood vessels to sites of excretion, mainly the liver and kidneys. The vasculature also enables inter‐organ communication and regulates the immune responses to infection and disease by distributing hormones, cytokines, and immune cells (leukocytes) from sites of production to target tissues.

**FIGURE 1:**
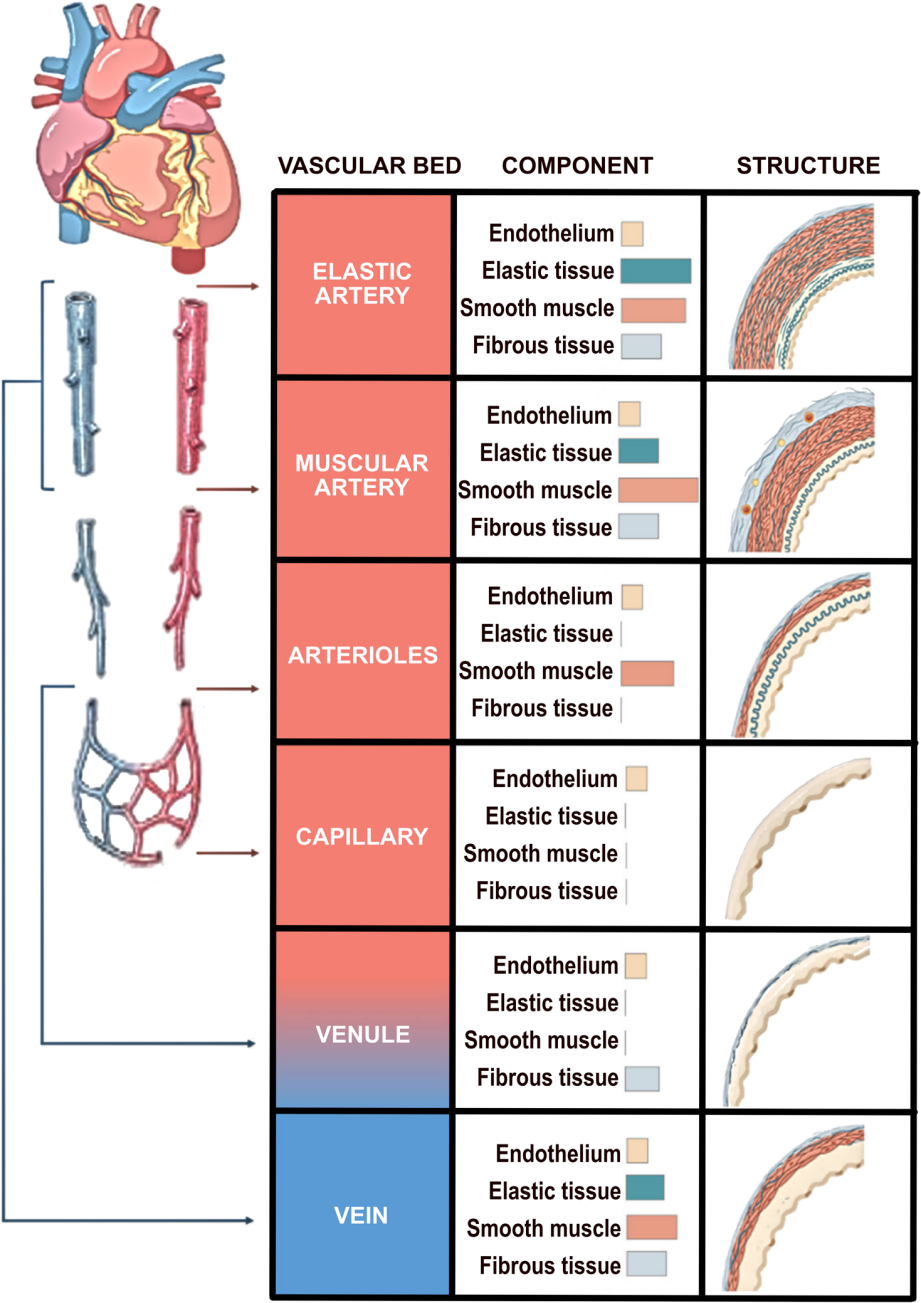
Structure of vascular system. Comparison of the walls of an elastic artery, muscular artery, arteriole, capillary, venule, and vein is shown. Licensed under CC BY 4.0 with modifications from reference (Martinez‐Martinez et al. 2021).

The lumen (inner space) of blood vessels is lined by endothelial cells (ECs) that are in direct contact with the bloodstream (Figure 1). In larger vessels, the basal surface of the endothelium is in contact with the inner elastic lamina (composed of extracellular matrix), also referred to as a basement membrane. In resistance arteries, ECs are also in direct contact with smooth muscle cells (SMCs) at a signaling complex known as myoendothelial junctions (MEJs), which has the capacity for intercellular gap junctional communication and regulation of nitric oxide (NO) signaling (King et al. 2022; Shu et al. 2019). Arteries have more smooth muscle than veins, reflecting their ability to dynamically alter vessel diameter. By contrast, capillaries and small veins (venules) that more subtly control vessel diameter lack SMCs and instead are supported by pericytes. In some tissues, small vessels also are in contact with specialized cells. This can include capillary ECs in contact with adipocytes (Luse et al. 2024), and astrocytes in contact with cerebral capillaries (Manu et al. 2023).

ECs have several roles that control major functions of the vasculature that can be impacted by disease. This includes (1) regulating solute and gas permeability and cell transmigration between the bloodstream and extravascular tissue, (2) regulating the rate of blood flow (via changes in vascular tone of resistance vessels and thereby blood pressure), and (3) repair/remodeling of injured vascular beds. These functions are exquisitely tissue‐specific and imply an intrinsic heterogeneity of endothelial phenotypes.

For instance, the liver and kidney microvasculature endothelium are highly permeable, reflecting the roles for these organs in blood filtration and removal of waste products. Liver sinusoidal EC monolayers have major discontinuities and little, if any, basement membrane reflecting a surface that enables unrestricted diffusion of material across the EC surface (Cleuren and Molema 2023). By contrast, glomerular ECs in the kidney are associated with a basement membrane and are fenestrated, allowing selective passive diffusion of substrates, mainly small molecules, salts and water. On the other hand, the blood brain barrier and pulmonary capillaries require ECs that form an exceptionally tight barrier to maintain tissue integrity and where gas exchange is mediated by erythrocytes in direct contact with vascular ECs (Borek et al. 2023; Eltanameli et al. 2024).

Given the important role for ECs in control of vascular permeability and vascular tone, the inability of the endothelium to perform these physiologically critical functions can have detrimental effects and results in a broad condition termed endothelial dysfunction (ED). Targeting the mechanisms of ED therefore represents a tantalizing pharmacological target for treatment of a large range of cardiovascular diseases. Control of blood pressure can be systemic, but it is also regulated at the level of individual organ systems. However, each vascular bed independently regulates arterial resistance and vessel permeability according to a tissue's physiological requirement, including high or low metabolic activity, transport of lipids, or removal of waste products. Given that the pathological consequences of ED are tissue bed specific, this indicates that pharmacological intervention should be specifically targeted.

### 1.1 Transcriptomics

**FIGURE 2:**
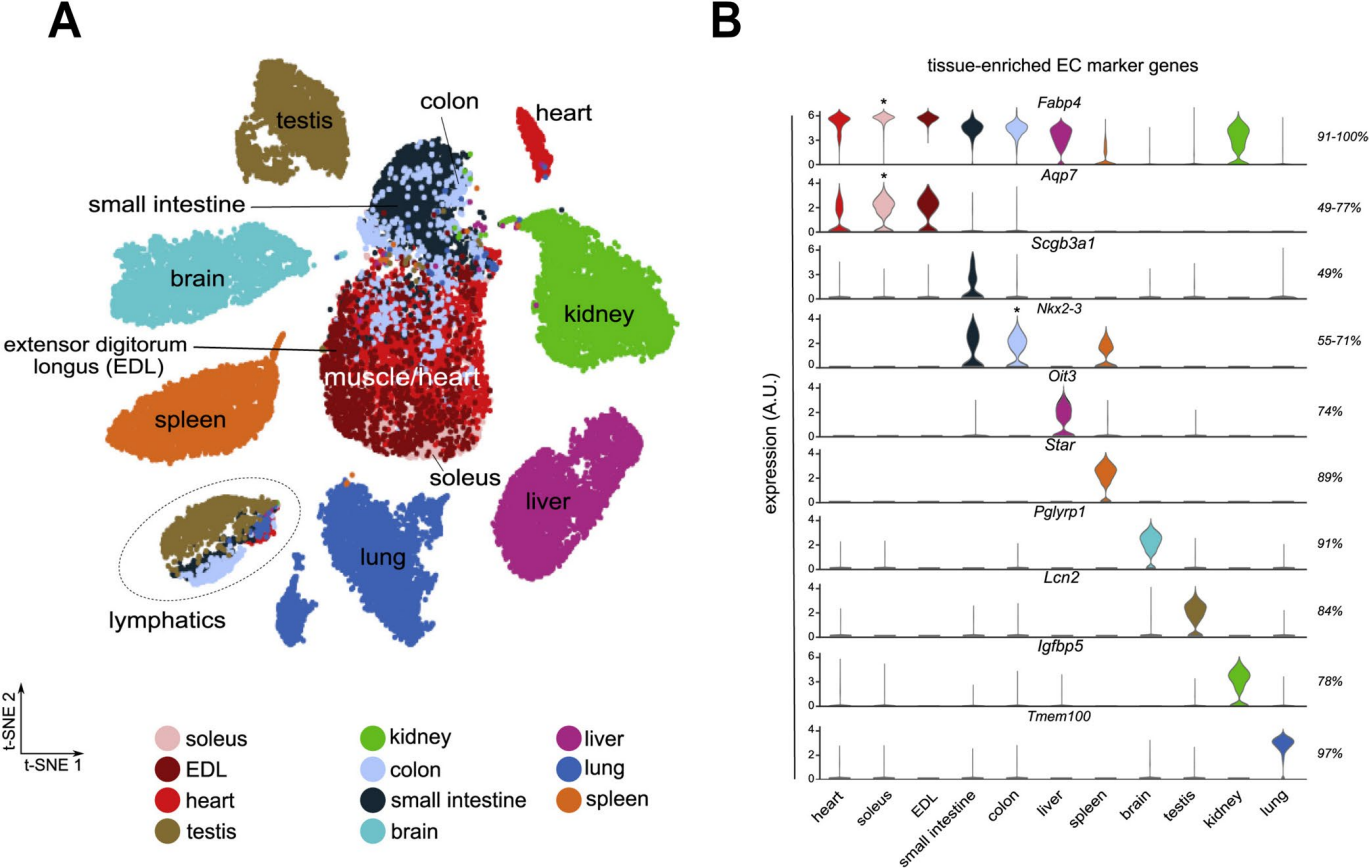
Tissue‐Specific Heterogeneity of endothelial cells. (A) t‐SNE plot of in silico‐selected ECs, color coded for tissue type. (B) Violin plots of the expression of markers, highly expressed in a substantial fraction (49%–100%) of ECs in the indicated tissues (% indicated on the right; in case of marker gene enrichment in multiple tissues, the violin plot with the highest expression is indicated by an asterisk). Reproduced with permission from reference (Kalucka et al. 2020) with modifications.

Single cell RNA sequencing (scRNAseq) and single nuclear RNA seq (snRNAseq) transcriptomics have revolutionized the ability to gain a more fundamental and mechanistic understanding of animal physiology. Comprehensive atlases such as Tabula Muris et al. (2018) and Tabula Sapiens et al. (2022) have been valuable resources to understand tissue and cell heterogeneity in healthy mice and humans, but the scope of these large datasets limits their utility to understand the phenotype of more specialized cell types, such as ECs, which are underrepresented in whole organ datasets. To overcome this limitation, scRNAseq analysis of immunopurified or lineage tagged ECs have been used to enhance the resolution of EC heterogeneity within tissues (Figure 2). This includes the Single‐Cell Transcriptome Atlas of Murine ECs, which resolves ECs from 11 distinct tissues in healthy mice (Kalucka et al. 2020). This approach has identified markers that can be used to effectively identify subclasses of ECs that otherwise could not be distinguished. For instance, markers for brain ECs (*Pglyrp1*) and lung ECs (*Tmem100*) were identified that enable cell subpopulations to be isolated and have the potential to be used for pharmacologic approaches to target specific ECs.

Several other organ‐specific ECs datasets have been published in recent years, including lung (Rodor et al. 2022), mesentery (Dunaway, Luse, et al. 2024), adipose (Dunaway, Luse, et al. 2024), and kidney (Dumas et al. 2020). The more specific scope of these analyses enables more EC subtypes to be identified and has the added advantage of enabling healthy and pathologic tissues to be analyzed and compared. For instance, targeted analysis of lung ECs isolated from mice and humans with pulmonary arterial hypertension (PAH) determined that there was a subpopulation of *Car4*+ lung capillary cells that became apoptotic, pro‐migratory, and upregulated pro‐angiogenic genes as part of the pulmonary hypertension (PAH) pathology (Rodor et al. 2022). As another example, scRNAseq analysis of ECs from mesenteric and adipose tissue of mice fed a high‐fat diet revealed high‐fat diet decreased the endothelial heterogeneity between these two tissues (Dunaway, Luse, et al. 2024). Specifically, high‐fat diet reduces the expression of genes encoding electron transport chain proteins in adipose arterial and capillary ECs, such that they become more similar to mesenteric arterial and capillary ECs. This is in alignment with decreased peroxisome proliferator‐activated receptor γ (PPARγ) expression in the adipose endothelium, suggesting a mechanism for a diabetic metabolic shift due to an obesogenic diet that is potentially targetable using PPARγ agonists (Dunaway, Luse, et al. 2024). These results were further validated by analysis of ECs from human adipose tissue, which showed a comparable effect correlating obesity with decreased PPARγ activity (Dunaway, Luse, et al. 2024).

### 1.2 Multi‐Cellular In Vitro Models

In vitro models of human ECs provide a strategy to translate basic science into human clinical relevance. However, monocultures of ECs are limited in their ability to fully model in vivo biology, which is influenced by their native tissue localization, enabling intercellular communication with other cell types. For example, resistance artery physiology depends upon direct contact between ECs and SMCs (King et al. 2022; Shu et al. 2019). Heterocellular EC:SMC contacts that faithfully mimic those found in resistance arteries can be produced using a vascular cell co‐culture model using Transwell permeable supports, which enable their molecular function to be analyzed (Biwer et al. 2017; Straub et al. 2012; Biwer et al. 2016).

It has been shown that the transcriptomic landscape of cells cultured in model systems is influenced by environmental factors such as cell–cell contacts and shear stress (Afshar et al. 2023). Organoids and organ‐on‐a‐chip models offer an approach to help recreate the native in vivo context in an in vitro model (Eltanameli et al. 2024). These systems have been used to investigate the contributions of endothelial function to tissue health in processes such as the regulation of immune cell migration, maintenance of barrier integrity, and predictions of transvascular drug permeability (Naderi‐Meshkin et al. 2023; Shelton 2024). Modeling the effects of flow on endothelial cell behavior has been particularly successful using models containing microfluidic systems (Ariyasinghe et al. 2025).

Although in vitro model systems have increased in sophistication, they are not without limitations. For instance, in vitro models typically approximate, but do not fully recapitulate native tissue architecture. This is particularly an issue when considering cell type multiplicity. Models containing one or two different types of cells (e.g., endothelial and smooth muscle cells) are relatively feasible to establish; however, incorporating three or more cell types into an in vitro model poses challenges. For instance, propagating different cell types while maintaining their phenotype usually requires different specific culture conditions that may not be compatible. One workaround that has been used is to substitute immortalized cell lines for one component of a given model system (Huh et al. 2010), with the caveat that cell lines may only partially represent primary cell phenotype.

Modeling cell substrates also poses challenges. It is most straightforward to synthesize organ‐on‐a‐chip models with stiff plastic substrates (Young's modulus > 1000 kPa) that can alter cell behavior. This can be partially ameliorated by precoating surfaces with extracellular matrix components prior to cell seeding (Staples et al. 2025). As a further refinement, organ‐on‐a‐chip models using hydrogels as cell substrates with Young's modulus < 1 kPa are currently being developed. As one example, a chip model using cells cultured on a collagen I/fibrin‐based hydrogel has been used to measure the impact of neuroblastoma on endothelial cell phenotype (Villasante et al. 2024). Regardless of model platform, an important caveat is that results obtained with in vitro systems should be validated in studies using native tissues.

## 2 Redox Signaling in Endothelial Function and Dysfunction

### 2.1 Classes of Reactive Species

Oxidants present in cells and tissues may be derived from external sources but are mainly generated from intracellular oxygen metabolism. Physiologically relevant oxidants include superoxide anion radical (O_2_^•−^), hydrogen peroxide (H_2_O_2_), and hydroxyl radical (^•^OH), all of which are produced as a result of mitochondrial respiration (Sies et al. 2022) and as enzymatic products of Nicotinamide adenine dinucleotide phosphate oxidases (NOXs) and other enzymes (Dustin et al. 2023). These were considered for a long time as simply byproducts of oxygen metabolism and defined as “reactive oxygen species” (ROS). Reactive species are not limited to oxygen radicals or H_2_O_2_. For instance, the predominant free radical species implicated in vascular function is nitric oxide (^•^NO). In the endothelium, NO is enzymatically produced by endothelial and inducible nitric oxide synthase (NOS) or by reduction of nitrite from heme proteins including alpha‐hemoglobin (Keller et al. 2022). Other reactive species contributing to vascular endothelial function are derivatives of hydrogen sulfide, produced endogenously from three enzyme classes and metabolized in mitochondria (Kolluru et al. 2023).

**FIGURE 3:**
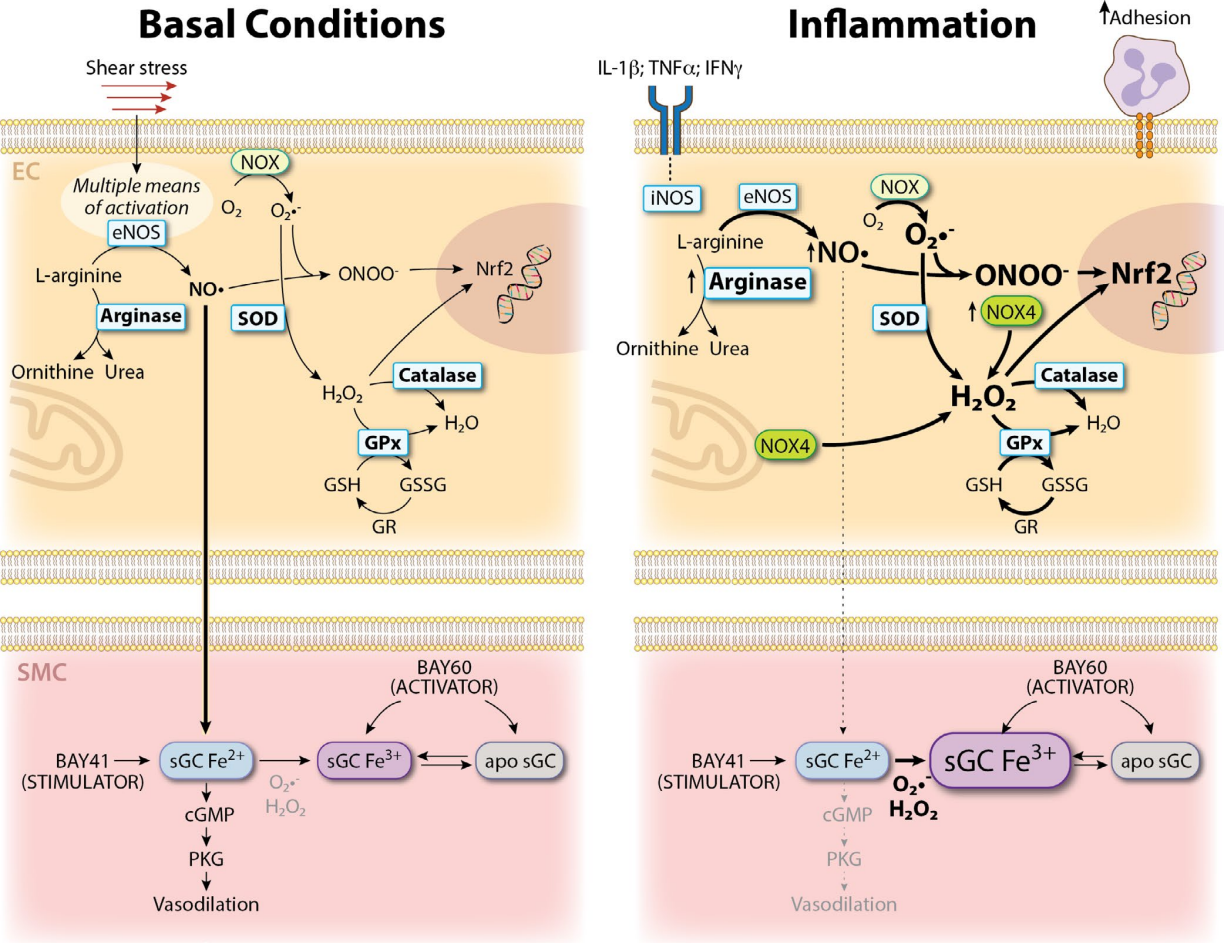
Comprehensive view of oxidative stress during endothelial dysfunction.

Decades of research have revealed that reactive species are not simply byproducts of oxygen metabolism leading to oxidative stress and vascular damage, but they are signaling molecules controlling endothelial function and tone (Sies et al. 2024). Reactive species are produced in a controlled fashion by specific enzymes, and interact with each other and defined cysteine targets on proteins, a system that was defined as a reactive species interactome (Cortese‐Krott et al. 2017). As demonstrated in Figure 3, there is significant regulation in homeostatic and pathological states of reactive species.

### 2.2 Endogenous Endothelial Antioxidant Responses

ECs have multiple antioxidant enzymatic systems optimized to protect them against oxidative damage. A well‐characterized pathway that fully detoxifies O_2_^•−^ involves mitochondrial and cytoplasmic superoxide dismutase, which converts O_2_^•−^ into H_2_O_2_, followed by catalase and glutathione peroxidase (GPx) that convert H_2_O_2_ into H_2_O. GPx uses the tripeptide glutathione as a cofactor for the reduction of H_2_O_2_, converting glutathione (GSH) into its oxidized form GSSG (glutathione disulfide), which is then converted back to GSH by glutathione reductase (see Figure 3). Further redox couples in the endothelium are thioredoxin and thioredoxin reductase, peroxiredoxin and glutaredoxin with their respective reductase systems. These protective proteins are specialized enzymes that detoxify peroxides and maintain thiols in a reduced form (Muhlenhoff et al. 2020; Gencheva et al. 2022).

The Nrf2/Keap1 system is also a fundamental antioxidant mechanism that has been discussed as a potential pharmacological target in ED (Itoh et al. 1997; Murakami et al. 2023). Nrf2 is a transcription factor that is cytosolically localized when complexed to Keap1. Stimuli such as shear stress‐induced NO or H_2_O_2_ release cause oxidation of cysteine residues in Keap1, which causes it to dissociate from Nrf2, enabling Nrf2 to translocate into the nucleus where it binds to antioxidant responsive elements that activate the expression of antioxidant genes, including glutathione synthetase, glutathione reductase, and heme oxygenase. The ability to activate a long‐term antioxidant response in ECs and other cell types has made Nrf2 an attractive pharmacological target for activators. Moreover, Nrf2 inhibitors have also been explored as potential anticancer drugs (Cuadrado et al. 2019). Polyphenols, sulforaphane, and other redox compounds present in food have the potential to activate Nrf2, although the dosages required for efficacy require application of purified agents with a rather limited therapeutic dose interval (Houghton et al. 2016). This and other complications related to targeted dosing and pleiotropic effects have limited the ability to target endothelial Nrf2 by pharmacologic agents (Cuadrado et al. 2019; Dayalan Naidu and Dinkova‐Kostova 2020).

### 2.3 NOS, Arginase and Soluble Guanylate Cyclase

The enzymes eNOS and iNOS are responsible for most EC NO production through the oxidation of L‐arginine into L‐citrulline. NO has multiple functions depending on the site of synthesis and on the targeted pathway (Farah et al. 2018; Lundberg and Weitzberg 2022). A fundamental role of NO produced by eNOS in endothelial function is best exemplified by its discovery as endothelium‐derived relaxing factor, having a central role in regulating vascular tone and therefore blood pressure. Specifically, low, tightly controlled, NO production by eNOS is necessary for control of vascular tone in response to shear stress and vasodilators. Underscoring the role of eNOS in regulating vascular tone, EC‐specific eNOS knockout mice lack the ability to respond to acetylcholine, have impaired flow‐mediated vasodilation, decrease coronary flow and bradykinin response in the heart, increased blood pressure, and impaired tissue perfusion (Leo et al. 2021; Cortese‐Krott et al. 2022; Dunaway, Saii, et al. 2024). As demonstrated in Figure 3, in contrast to constitutively expressed eNOS, which has a central role in baseline endothelial function, iNOS is induced in response to inflammatory stimuli via NF‐κB activation where it can have a protective effect in acute inflammation or a damaging effect in chronic inflammation (Kim and Lee 2025; Carlstrom et al. 2024).

Although eNOS is constitutively expressed by endothelial cells, it is also a highly polymorphic gene with over 2000 different human genetic isoforms being identified ranging from single nucleotide polymorphisms (SNPs) to gene insertions/deletions (Oliveira‐Paula et al. 2017). These alterations in the gene can have a significant impact on cardiovascular function and disease. For example, polymorphisms in the eNOS (NOS3) promoter have been found to alter the affinity of ETS family transcription factor binding, resulting in different levels of eNOS protein and functionality that can impact the development of hypertension (Salvi et al. 2013). Another example is the Glu298Asp polymorphism associated with multiple cardiovascular diseases (Rai et al. 2023). This single amino acid shift diminishes the ability of eNOS to bind to caveolin‐1, reducing eNOS activation and, thus, circulating NO levels (Joshi et al. 2007). The functional ramifications of these and other eNOS alleles underscore the importance of both genetic and environmental factors in considering causative factors that cause ED.

Arginine is an immediate precursor to NO, so it has been proposed that levels of EC NO could be controlled either by arginine or citrulline supplementation, or by inhibition of arginase, the enzyme catalyzing the degradation of arginine into urea (Caldwell et al. 2015). Studies attempting these approaches proved ineffective, since arginine metabolism is controlled mainly by the liver and the kidney (Heuser et al. 2025; Mahdi et al. 2020). Thus, the best pharmacological approaches to manipulate NO‐dependent pathways are focused on targeting specifically the NO receptor soluble guanylyl cyclase (sGC). NO activates sGC by reacting with the Fe^2+^ heme in the beta subunit (Wittenborn and Marletta 2021). In some disease states, oxidation of the Fe^2+^ heme into Fe^3+^ heme inhibits the ability of sGC to bind NO, eventually leading to loss of the heme and sGC protein degradation. Two independent classes of drugs can target sGC (Sandner et al. 2021): (1) stimulators (such as Vericiguat) which synergistically work with NO to enhance sGC signaling and (2) activators (such as Cinaciguat) which activate the oxidized Fe^3+^ sGC or the apo (heme free) sGC in an NO‐independent manner (Sandner et al. 2021; Stasch et al. 2006; Evgenov et al. 2006; Liu et al. 2021).

So far, only stimulators have been approved for clinical use. For instance, sGC stimulators have shown efficacy in treating diseases less impacted by oxidative stress, such as chronic heart failure with reduced ejection fraction (HFrEF) (Armstrong et al. 2018; Kang and Lamb 2022). Although sGC activators are not currently approved for use in humans, they have been shown to have efficacy in a mouse model of NO‐resistant coronary artery disease (Stasch et al. 2006). Having pharmacologic agents that target different root causes of ED extends the potential to tailor treatment regimens.

### 2.4 NOX Enzymes

NOXs catalyze the conversion of oxygen into O_2_^•–^ or H_2_O_2_, depending on the NOX isoform (Figure 3) (Kracun et al. 2025). Of the seven NOX isoforms, four are expressed in the vascular endothelium (NOX1, NOX2, NOX4, and NOX5) and play distinct roles in regulating cell behavior in response to intracellular or extracellular stimuli. NOXs have been identified as playing a central role in processes such as cell migration, proliferation, and cytoskeletal organization (Zhang et al. 2020). However, increased NOX activity can significantly contribute to the pathogenesis of many cardiovascular diseases. Excess production of O_2_^•–^ or H_2_O_2_ through NOX‐dependent mechanisms is thought to lead to eNOS oxidation, monomerization, or uncoupling, and to sGC oxidation, promoting ED and ultimately contributing to conditions such as heart failure and hypertension (Loperena and Harrison 2017; Daiber et al. 2021).

Among the NOXs expressed by ECs, NOX4 is unique due to its constitutive generation of H_2_O_2_ as opposed to other NOX isoforms, which depend on superoxide dismutase to convert O_2_^•–^ into H_2_O_2_. As an essential cell signaling molecule, H_2_O_2_ can increase eNOS expression and therefore, perhaps, NO production (Garcia‐Prieto et al. 2019). The expression of NOX4 in the context of hypertension has been repeatedly studied in several model systems (Ray et al. 2011; Bouabout et al. 2018) and results have proven to be enigmatic. However, recent data has demonstrated endothelial‐derived H_2_O_2_ via NOX4 (Wolpe et al. 2024), and direct chemical activation of H_2_O_2_ induces hypertension (Das et al. 2025). H_2_O_2_ is also a well‐used model system to induce smooth muscle phenotypic switching. Thus, it is likely that NOX4‐derived H_2_O_2_, chronically at least, is net negative in terms of blood pressure regulation. In relation to plaque progression and atherosclerosis, NOX4 activity has been shown to be both athero‐protective and detrimental to the vasculature (Gray et al. 2016; Lozhkin et al. 2017). Altogether, NOX4's role in aggravating or ameliorating the progression of ED is largely dependent on its locale as well as the specific disease or cell type. Development of isoform‐specific, spatially targeted NOX therapeutics allows for the modulation of ROS production and resulting downstream events that have been implicated as mechanisms leading to ED progression (Dustin et al. 2023).

## 3 Endothelial‐Dependent Pathophysiology in Different Vascular Beds

### 3.1 Coronary Artery

Coronary artery disease is due to a narrowing or complete blockage of blood flow to the heart caused by atherosclerosis, a buildup of cholesterol and other biomaterials to form a plaque. The endothelium plays several roles in the progression of atherosclerosis. In the early phases of the disease, ED exacerbated by inflammation causes increased expression of adhesion molecules, such as ICAM‐1, that recruit circulating monocytes to bind to the endothelium, ultimately migrating into the vessel wall where they differentiate into macrophages that phagocytose lipoproteins, narrowing the vessel lumen and progressively blocking blood flow (Libby et al. 2019). Significant vessel blockage can eventually result in myocardial infarction due to a loss of oxygen delivery to cardiac tissue.

Although implanting a vascular stent is a widely used treatment for atherosclerosis, spontaneous healing from a blocked artery requires revascularization, led by endothelial proliferation driving angiogenesis (Zhang et al. 2014). However, if the endothelium is sufficiently damaged, for example, by ischemia/reperfusion (I/R) injury, it can lose its reparative capacity and become fibrotic, often referred to as an endothelial to mesenchymal transition (EndoMT) (Kovacic et al. 2019; Alvandi and Bischoff 2021; Greaves and Calle 2022). One of the consequences of EndoMT is that the fibroblastoid cells deposit increased extracellular matrix, which stiffens the vessel and inhibits its compliance. EndoMT is particularly prevalent in endocardial and valve ECs, contributing to mitral valve thickening and calcification but may also occur in vascular ECs in the heart wall, contributing to cardiac fibrosis (Kovacic et al. 2019; Bartko et al. 2017). There are many signaling pathways that drive EndoMT with the potential to be targeted to reverse profibrotic ED, including notch signaling, vascular endothelial growth factor A (VEGFA), and transforming growth factor β (TGFβ) (Song et al. 2016; Li, Dong, et al. 2013; Rossato et al. 2020; Pinto et al. 2018). For instance, hepatocyte growth factor (HGF) has been found to decrease EndoMT induced by TGFβ and could be a potential therapeutic approach (Wang et al. 2018).

Recent work has focused on two natural alkaloids, matrine (MT) and oxymatrine (OMT), as potential therapeutics for cardiovascular disease by targeting the JAK–STAT pathway that is activated by TGFβ, thus protecting ECs from I/R injury (Ghasemi Pour Afshar et al. 2024; Guo et al. 2018). MT also suppressed the production of ROS, reducing EC apoptosis (Liu et al. 2017). Treatment with OMT has also been found to significantly prevent cardiac cell death and decrease secretion of pro‐inflammatory cytokines such as TNFα and IL‐6 (Sun et al. 2023). This is likely due to OMT‐mediated activation of the SIRT1/Nrf2 signaling pathway, enhancing expression of antioxidant proteins and inhibiting inflammation (see Section 2.2, above). This, in turn, inhibits NLRP3 inflammasome‐mediated pyroptosis, programmed cell death otherwise associated with release of pro‐inflammatory cytokines (Jin et al. 2021). Taken together, these alkaloids appear to be promising therapeutics for cardiovascular disease treatment and prevention, but their bioavailability is low, which limits their pharmacological efficacy (Sun et al. 2022; Li et al. 2023). Developing new MT and OMT‐related compounds with increased bioavailability is needed to increase the therapeutic potential of targeting these pathways to treat cardiovascular disease.

Since atherosclerotic lesions have been found to contain an excess of iron, this suggests that ferroptosis is another pathway with the capacity to exacerbate disease severity by inducing EC death (Sullivan 2009; Marques et al. 2019; Xu 2019). Consistent with this possibility, inhibition of ferroptosis using ferrostatin‐1 reduces the progression of atherosclerosis in mice fed a high‐fat diet and is associated with suppressing lipid peroxidation, which otherwise damages EC function (Bai et al. 2020). This also suggests that combined targeting of pyroptosis and ferroptosis might have the best efficacy in targeting atherosclerotic plaques.

### 3.2 Renal Endothelium and Filtration

Chronic kidney disease (CKD) is defined by kidney damage or reduced filtration capacity for a time > 3 months that impacts overall health (Inker et al. 2014). Patients with moderate to advanced CKD have an increased cardiovascular risk compared to the general population, primarily due to impaired glomerular filtration (Emini Veseli et al. 2017). Renal filtration capacity is due in large part to the highly diversified kidney vasculature that has significant intercompartmental heterogeneity. At least 24 transcriptionally different EC populations have been identified (Dumas et al. 2020). These can be broadly grouped into three compartments that are needed for proper filtration of the molecules and ions circulating in the bloodstream: the glomeruli, cortex, and medulla (Savige 2014). Each of the three compartments has its own unique EC populations, which differentiate in response to cues depending on different local microenvironments.

Of these different classes of ECs, glomerular ECs form a critical monolayer that regulates filtration of the bloodstream into the proximal tubule and ultimately, urine. Glomerular EC dysfunction is a critical factor in the development of progressive kidney diseases (Reidy and Kaskel 2007; Fioretto and Mauer 2007). For instance, in animal models of glomerular necroptosis associated with glomerular EC inflammation, the disease can be suppressed by inhibiting NF‐κB with bortezomib. A more specific approach using E‐selectin targeted liposomes to deliver p65 specific siRNAs to inflamed ECs also has some efficacy (Choi et al. 2017).

Alport syndrome is a rare inherited disease resulting from a mutation in one of the collagen VI genes (most frequently *COL4A5*), which induces changes in the overall composition of the glomerular basement membrane (GBM). This, in turn, can impact glomerular EC and podocytes, further disrupting the GBM, causing increased mechanical strain, which results in tissue fibrosis and a proinflammatory environment (Savige 2014). Eventually, this leads to proteinuria due to a pathologic increase in glomerular EC permeability. This pathology has been recapitulated in an Alport syndrome mouse model analyzed using intravital imaging to detect glomerular damage undetectable in standard renal histological analysis (Gyarmati et al. 2022). Patients with Alport syndrome also are subject to hypertension, which further exacerbates the severity of the disease by increasing mechanical stress in the glomerulus. Consistent with a role for hypertension in the progression of Alport syndrome, angiotensin‐converting enzyme (ACE) inhibitors have been used in a patient cohort and proven to be an effective therapeutic approach, especially when administered early in lifespan (Boeckhaus et al. 2022).

### 3.3 Bone Marrow Niches and Hematopoiesis

As a site of stem cell maturation and hematopoiesis, bone marrow ECs (BMECs) play a pivotal role in the architecture of specialized bone sinusoidal niches (Lucas 2021). These are microenvironments in the bone marrow that are crucial for hematopoietic stem cell (HSC) function, required for the development of a broad range of immune and non‐immune cell types, including erythrocytes, leukocytes, and thrombocytes (Rafii et al. 1995). HSCs express integrins with the capacity to bind to EC vascular cell adhesion molecule 1 (VCAM‐1), consistent with direct interactions between HSCs and BMECs. In addition to providing a structural niche, BMECs also provide hormonal cues such as stem cell factor and C‐X‐C motif chemokine 12 (CXCL12) that regulate HSC differentiation and proliferation (Kwon et al. 2024).

Consistent with a key role for BMECs in the control of HSCs, cardiovascular diseases including hypertension, atherosclerosis, and myocardial infarction have been shown to cause excess HSC differentiation which can exacerbate disease, suggesting that BMECs are sensitive to systemic ED (Rohde et al. 2022). Vascular complications related to diabetes can also impact BMEC function. This was recently examined in three different mouse models of diabetes where it was found that BMECs produced less CXCL12 which impaired signaling via a novel EC specific epithelial growth factor receptor (EGFR) pathway required to maintain HSC quiescence (Hoyer et al. 2020). Recently, siRNA‐loaded nanoparticles were developed that can target and alter BMEC function in vivo (Krohn‐Grimberghe et al. 2020; Sago et al. 2018). Using this platform, it is possible to deliver siRNAs that decrease the expression of factors that either inhibit or enhance HSC differentiation and release into the circulation. Therefore, this may be a potential therapeutic option to normalize hematopoiesis under conditions where it would be otherwise impaired in response to cardiovascular disease (Krohn‐Grimberghe et al. 2020).

Acute graft‐versus‐host disease (aGvHD) is a significant complication of hematopoietic stem cell transplantation (Kwon et al. 2024; Mir et al. 2017). While the etiology of aGvHD is complex, disease severity is related to systemic EC damage as measured by levels of pro‐inflammatory markers present in the bloodstream, including VCAM‐1, intracellular adhesion molecule 1 (ICAM‐1), and TNFα receptor 1 (TNFR1). In fact, EC‐derived TNFR1 levels in serum have clinical significance as a diagnostic biomarker (Reikvam et al. 2024). Given the nature of the disease, aGvHD treatments have focused on systemic anti‐inflammatory therapeutics, including corticosteroids, biologics, and fecal microbiota transplant therapies to normalize the immune response instead of trying to directly repair the BMEC niche (Guo et al. 2024; Velickovic et al. 2020).

### 3.4 Perivascular and Visceral Adipose Tissue

There is accumulating evidence that perivascular adipose tissue (PVAT) may regulate endothelial function via the production of H_2_O_2_ and thereby contribute to the regulation of vascular tone (Gao et al. 2007). To test the effect of PVAT on vessel function, reactivity of healthy and diabetic rat aortas was assessed with and without intact PVAT (Azul et al. 2020). PVAT from healthy rats' phenylephrine‐mediated constriction; however, this effect was lost in diabetic rats. PVAT from diabetic rats showed increased inflammatory markers, as well as decreased expression of superoxide dismutase and catalase. Vessels from diabetic rats had improved acetylcholine‐induced vasodilation when treated with catalase mimics like tempol and/or by inhibiting inflammatory toll‐like receptor‐mediated response by using the inhibitor CLI‐095. Additionally, aortic PVAT where PVAT‐free aortas from mice fed a high‐fat high‐sucrose (HFHS) fed diet showed no difference in endothelial‐dependent relaxation as compared to mice fed control chow diet. However, when PVAT was left on aortas from mice fed high‐sucrose high‐fat diet, it showed decreased vasodilatory capacity when compared to normal chow fed controls (Osaki et al. 2023). The diminished vasodilation seen with aortas containing PVAT from HFHS fed mice was rescued with the use of tempol, indicating that these effects were mediated by O_2_^•−^ and/or H_2_O_2_ formation by PVAT. These effects appear to be mediated by NOXs. Indeed, NOX1, NOX2, and NOX4 are all expressed by PVAT (Quesada et al. 2018).

Conversely, visceral white adipose tissue can have detrimental effects on cardiovascular disease outcomes in obesity as oxidative stress and inflammation increase immune infiltration and adipokine release (Koenen et al. 2021; Kunz et al. 2021). This oxidative stress can also promote the proliferation and differentiation of preadipocytes as well as hypertrophy of mature white adipocytes (Baldini et al. 2021). In turn, increased adipocyte growth negatively impacts the vasculature, causing endothelial dysfunction.

To assess the role of non‐PVAT white adipose tissue in oxidative stress‐induced endothelial dysfunction, human thoracic adipose tissue samples were analyzed (Akawi et al. 2021). It was found that an increased volume of thoracic adipose tissue was associated with increased arterial oxidative stress. High thoracic adipose tissue sphingolipid secretion was also significantly associated with a reduction in EC NO bioavailability as well as increased O_2_^•−^ generation detected by the lucigenin assay. Circulating C16:0‐ceramide was positively correlated with O_2_^•−^ production and increased systemic inflammation in over 600 patients with atherosclerosis. Additionally, patients given the glucagon‐like peptide analog liraglutide saw a marked reduction in ceramide secretion in obese patients. This shows liraglutide or other similar medications to be a potential treatment for adipose‐induced vascular dysfunction by targeting ceramide release from adipocytes themselves.

### 3.5 Cerebral Vasculature, Cognitive Decline and Stroke

The endothelium in the brain is integral to regulating tissue perfusion and barrier function. Specifically in the cerebral vasculature, the endothelium forms more restrictive junctions contributing to a highly selective blood‐brain barrier (BBB) that protects the brain from direct exposure to potentially harmful blood components. Tight junctions of ECs in the BBB are essentially impermeable. Instead, cerebral ECs have transporters that mediate the transfer of nutrients and metabolic waste products between the CNS and the circulation (Zhao et al. 2015). In mice, it has been shown that transporter function is regulated by circadian rhythms, where efflux from the CNS tends to be favored during the night cycle, when these nocturnal animals are most active (Zhang et al. 2021).

Transcytosis also plays a key role in regulating plasma protein transport across the BBB (Yang et al. 2020). Interestingly, there is an aging‐associated shift from receptor‐specific transcytosis driven by clathrin‐mediated endocytosis to non‐specific caveolar transcytosis. This shift in specificity is associated with an increase in alkaline phosphatase activity. Critically, the balance of specific to non‐specific transcytosis can be restored by intravenous administration of a specific small molecule alkaline phosphatase inhibitor (compound 613,810), suggesting a potential therapeutic approach to treat age‐related impairment of the BBB (Yang et al. 2020).

Cerebral vessel blockage leads to stroke. While it is difficult to directly measure cerebral endothelial function in humans, studies have shown that impaired flow‐mediated dilation, a measure of endothelial function, is associated with more severe strokes and worse outcomes (Santos‐Garcia et al. 2009). In mice, endothelial‐specific deletion of the channel‐forming protein pannexin‐1 attenuated inflammation associated with vessel ischemia, leading to less severe stroke and suggesting that pannexin‐1 inhibitors such as spironolactone will have therapeutic utility (Good, Chiu, et al. 2018; Good, Eucker, et al. 2018; Li, Atochin, et al. 2013).

ED due to diabetes is another risk factor for stroke and cognitive decline (Good, Eucker, et al. 2018; Li, Atochin, et al. 2013; Edgerton‐Fulton and Ergul 2022). Of note, diabetic mice show increased cerebral vessel density and tortuosity, as well as decreased vessel diameter and deficiency in pericytes (Li et al. 2010). These anatomical changes can render cerebral vessels more prone to cerebral ischemia. The resultant local hypertension in the cerebral vasculature can exacerbate the severity of stroke (Good, Eucker, et al. 2018; Li, Atochin, et al. 2013). However, the cerebral vasculature can be partially normalized by treatment with metformin.

### 3.6 Pulmonary Vasculature

ED has been proven to be both a cause and consequence of diseases associated with lung remodeling in response to injury, such as pulmonary arterial hypertension (PAH), chronic obstructive pulmonary disease (COPD), and interstitial lung disease (Rydell et al. 2018; Kurakula et al. 2021). ED associated with COPD causes a loss of alveolarization, which can significantly impair gas exchange by reducing the surface area of the respiratory zone of the lung (Ravi et al. 2024). Underscoring the importance of ECs in driving lung structure, intravenous administration of healthy alveolar ECs to mice with emphysema restored the structure and function of the respiratory zone, suggesting a potential therapeutic approach (Hisata et al. 2021).

As another example, in PAH, factors such as EC injury, shear stress, and hypoxia lead to an overall proinflammatory shift in the pulmonary endothelium associated with conducting airways (Haensel and Wojciak‐Stothard 2023). Although the exact mechanisms contributing to ED in PAH can vary, remodeled pulmonary arteries are the ultimate consequence. This shift towards ED can then trigger SMC hyperproliferation, which exacerbates pathologic vasoconstriction, leading to increased blood pressure that puts significant stress on the heart (Bousseau et al. 2023). A specific EC‐targeted strategy to combat the progression of vessel hypermuscularization after the onset of ED could have therapeutic value, especially if it was administered early enough; however, it would have limited efficacy unless it can also renormalize the SMC layer (Haensel and Wojciak‐Stothard 2023).

In addition to cellular integrity, the lung also must maintain an air‐liquid interface to enable efficient gas exchange. Failure of this barrier results in a condition called acute respiratory distress syndrome (ARDS). The air‐liquid interface is maintained by the barrier function of both the endothelial and epithelial lung tissue layers (Schlingmann et al. 2015). The EC barrier is particularly critical and can be impaired by several insults, including inflammation associated with sepsis. Of particular relevance, EC pannexin‐1 channels have been shown to mediate failure of venous EC barrier function in response to the pro‐inflammatory cytokine TNFα (Maier‐Begandt et al. 2021). The pannexin‐1 inhibitor, spironolactone, reduces the severity of sepsis in mouse models, suggesting that pannexin‐1 may have efficacy as a therapeutic approach.

## 4 ECs and Interorgan Crosstalk

ECs are systematically interconnected via the cardiovascular system, which enables their function to be systemically coordinated. A classic example of this is endocrine signaling to control blood pressure, which requires coordination of factors produced by the liver (angiotensinogen), kidney (renin), and pulmonary vasculature (ACE) that are secreted into the vasculature forming angiotensin I and angiotensin II that have differential effects on different target tissues. This enables systemic blood pressure, regulated mainly by resistance arteries, and local renal blood flow to be independently controlled. Metabolic control of blood glucose levels by insulin and glucagon is another well‐studied example where systemic coordination of multiple organs interconnected through the vasculature is required for control of metabolism, glucose partitioning, and energy production.

More recently, it has become appreciated that extracellular vesicles (EVs) can provide another mode of inter‐endothelial communication that can be either beneficial or deleterious (Jansen et al. 2017). EVs are membrane vesicles secreted from cells that have specific surface compositions, including transmembrane receptor proteins, and can be enriched for sphingolipids. Within their lumen, different classes of EVs can contain a range of bioactive molecules ranging from cytokines to coding and noncoding RNAs. Because of their biological specificity, EVs can mediate communication between ECs in different vascular beds by virtue of their site of origin, trafficking through the vasculature, and binding to receptors of target ECs. Once internalized, EVs release their contents, which can alter cell behavior, providing an effective pathway for the transfer of bioactive molecules from one set of ECs to another. However, it is important to note that most of the current knowledge of the functional effects of EVs has been identified from examining the effects of purified, concentrated EVs harvested from cells and applied as a bolus to a specific target cell in vitro or administered to animals. Less is known about endogenous pathways mediated by the release of physiological levels of EVs.

Functionally, endothelial EVs have been found to promote endothelial repair by several pathways. For instance, miRNA delivered to target cells has been found to promote EC repair of denuded vessels in situ, an effect that was abrogated by exposing EVs to high glucose, suggesting that this repair pathway is impaired in diabetes mellitus (Jansen et al. 2013). Conversely, there are also pools of EVs that can have a deleterious effect on EC function. Several studies have demonstrated that endothelial EVs can impair regulation of blood pressure by inhibiting NO production in target ECs (e.g., (Good et al. 2020)). For instance, EVs have caused a significant decrease in endothelium‐mediated vasodilation, an effect that was also associated with an exacerbation of acute lung injury (Densmore et al. 2006). Thus, EVs represent a double‐edged sword with respect to vascular health and disease, but ultimately are likely to provide specific therapeutic options to promote EC function and repair, both as a target and as a clinically administered therapeutic approach.

## 5 Concluding Remarks and Future Perspectives

Next generation transcriptomics has revealed a remarkable heterogeneity in EC phenotype and responses to disease. ECs are more than an inert lining of the vasculature; instead, they act as sensors and regulators of vascular status that act in concert with other cell types present in the vasculature. Thus, ED has a significant impact on cardiovascular health and pathophysiology. Major causes of ED include oxidative and mechanical damage, as well as mechanical disruption of EC function, such as altered blood flow or increased tissue stiffness. Current therapeutic strategies have focused on identifying pharmacologic agents that target enzymes and receptors specifically present in EC subpopulations. While systemic application of pharmacologic agents has demonstrated efficacy in the treatment of cardiovascular disease, there is still room to engineer additional specificity into therapeutic approaches. For instance, multi‐component nanostructured particles designed to target epithelia (Hansen et al. 2024) could be modified to offer the potential to target EC subpopulations expressing distinct cell surface molecules to deliver bioactive compounds to precise sites impacted by ED. Developing in vitro platforms that faithfully mimic native healthy and diseased in vivo niches that are amenable to high throughput screening would help identify innovative approaches to identify the most efficacious methods to treat cardiovascular disease.

## Conflicts of Interest

The authors declare no conflicts of interest.

## Data Availability

The authors have nothing to report.
